# Target localization for post‐prostatectomy patients using CT and ultrasound image guidance

**DOI:** 10.1120/jacmp.v6i4.2137

**Published:** 2005-11-22

**Authors:** Kamen Paskalev, Steven Feigenberg, Rojymon Jacob, Shawn McNeeley, Eric Horwitz, Robert Price, Charlie Ma, Alan Pollack

**Affiliations:** ^1^ Department of Radiation Oncology Fox Chase Cancer Center 333 Cottman Avenue Philadelphia Pennsylvania 19111 U.S.A.

**Keywords:** post‐prostatectomy, localization, organ motion, CT, ultrasound

## Abstract

We conducted a study comparing B‐mode acquisition and targeting (BAT) ultrasound alignments based on CT data in the postoperative setting. CT scans were obtained with a Primatom CT‐on‐rails on nine patients. Two CT scans were obtained each week, while setup error was minimized by BAT ultrasounds. For the first three patients, a direct comparison was performed. For the next six patients, a template based on the shifts from the week 1 CT during treatment was used for subsequent setup. Comparison of isocenter shifts between the BAT ultrasound and CT was made by the difference, absolute difference, and improvement (using CT alignments as the reference technique). A total of 90 image comparisons were made. The average interfraction motion was 3.2 mm in the lateral, 3.0 mm in the longitudinal, and 5.1 mm in the AP direction. The results suggest that the CT‐based ultrasound templates can improve the localization of the prostate bed when the initial displacements are greater than 4 mm. For initial displacements smaller than 4 mm, the technique neither improved nor worsened target localization. However, ultrasound alignments performed without the use of a template deteriorated patient positioning for two out of three patients, demonstrating that the use of a CT template was beneficial even at small initial displacements.

PACS numbers: 87.53.‐j, 87.53.Kn, 87.53.Xd

## I. INTRODUCTION

Improvements in radiotherapy treatment techniques and delivery have led to improvements in treatment outcomes. Perhaps the best example of this is in the management of prostate cancer where the development of 3D conformal radiotherapy has improved freedom from biochemical failure due to the ability to safely escalate radiation dose.^(1)^ As doses are increased, the precision of dose delivery becomes crucial, so the treatment must be performed with great accuracy. However, daily uncertainties regarding patient setup reproducibility (i.e., daily setup error and internal organ motion) diminish the ability to achieve this goal.^(2)^ In the past, the frequently changing nature of these variations has made corrections difficult and dictated that a wider margin of normal tissue be included within the radiation field to ensure tumor coverage.

Various approaches have been used to reduce these uncertainties including daily imaging prior to treatment using abdominal ultrasound^(^ ^3^ ^–^ ^9^ ^)^ and CT scans,^(^ ^10^ ^–^ ^12^ ^)^ immobilization devices^(13)^ (i.e., rectal balloon), and placement of fiducial markers^(^ ^7^ ^,^ ^8^ ^,^ ^14^ ^–^ ^18^ ^)^ in the prostate gland. Unfortunately, the majority of the data with these devices are in the definitive setting where the target or prostate is well visualized. In the postoperative setting, the target is not easily visualized, which potentially reduces the effectiveness of these devices.

Currently at our institution, daily uncertainties are reduced with the use of the B‐mode acquisition and targeting (BAT) ultrasound system for definitive and postoperative patients. In October 2002, a CT was placed in the LINAC vault. The CT‐on‐rails system has been used, like the BAT ultrasound system, to reduce daily uncertainties.^(12)^ This report describes our initial experience in implementing this device in the postoperative setting.

## II. METHODS

In June 2003, a Primatom sliding CT gantry (Siemens Medical Solutions, Concord, CA) was first used for localization of a post‐prostatectomy patient at our institution. Since then, it became the department's policy to obtain CT scans twice per week to verify localization of post‐prostatectomy treatment volume for patients currently undergoing daily localization using BAT ultrasound system (Nomos Corporation, Cranberry Township, PA). This study describes our initial experience based on the first nine patients evaluated. All patients were treated in the supine position using an alpha cradle cast as previously described.^(19)^ Target volumes and critical normal structures were contoured using the combined fused images from the CT and MR simulations as previously described.^(20)^ CT and MR simulations were obtained using axial images obtained every 3 mm through the pelvis. The target volume was based on presurgery CT scans of the pelvis and the pathologic specimen in order to cover the prostatic fossa and periprostatic tissues, specifically including the bladder neck, anastomosis, seminal vesicles, and surgical clips. Some of the regional lymph nodes were included if the initial surgical lymph node dissection was not done or was considered inadequate. All patients were treated with intensity‐modulated radiotherapy (IMRT) to a total dose of 64 or 66 Gy to 70 Gy for either adjuvant or salvage, respectively. IMRT was planned using the Corvus treatment‐planning system (Nomos Corporation, Cranberry Township, PA).

To reduce organ motion and setup error, the BAT ultrasound system is used at our institution. The BAT ultrasound system calculates the daily shifts based on an alignment of cross sections of the volumes drawn on the simulation CT scan with ultrasound images of the target region in the treatment position. The procedure used to acquire a daily CT scan and to perform ultrasound‐based alignment is described as follows:

1. The patient is immobilized in a treatment position, and the skin marks from simulation are aligned with the room lasers. Radio‐opaque fiducial markers are placed on the skin. Markers coincide with the lasers and are used to define the daily isocenter in the localization CT scan.
2. The treatment table is rotated 180° (scanning position), and a CT scan is obtained with the moving gantry.
3. The table is rotated back to the treatment position.
4. Alignment of the fiducial markers with the room lasers is verified. If the fiducial markers have moved more than 1 mm during the scan, the displacement is recorded and is later taken into account when calculating the daily shifts. If the displacement is greater than 3 mm or one of the fiducials is not visible, the scan is discarded from the study.
5. BAT ultrasound alignment is performed, and the treatment table is shifted in the *x*, *y*, *z* direction prior to treatment.

**Figure 1:**
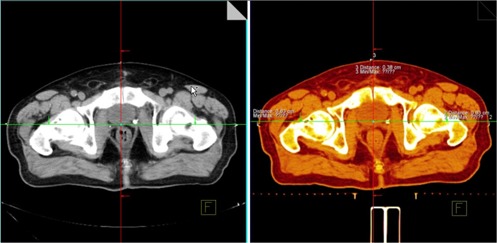
CT shift calculation. After the targets on the simulation and the localization CT scans have been aligned, the cross hair is positioned at the isocenter of the simulation scan (left). Then the distances between the cross hair and the fiducial markers in the localization scan give the CT shifts.

The pretreatment CT (i.e., localization CT) scans were fused with the simulation CT scan in order to calculate the daily CT shifts. The shifts were determined using the Coherence Dosimetrist workstation (Siemens Medical Solutions, Concord, CA). The main fusion landmarks were the posterior bladder wall and the anterior rectal wall. After the fusion, the daily CT shift was measured as the distance between the fiducial markers, which are visible on both simulation and localization scans (Fig. 1).

The CT shifts were assumed to be more accurate than the BAT ultrasound shifts and were used as the reference shifts. The uncertainty of our image fusions for the two CT scans was estimated to be 2 mm based on the 3‐mm slice thickness and associated pixel size. The uncertainty in the position of the fiducial markers was 1 mm. Therefore, the total CT shift uncertainty was estimated to be 2.5 mm. This was verified by testing both the intrauser and interuser variability of the CT shifts, which was within 2 mm (data not shown).

**Figure 2:**
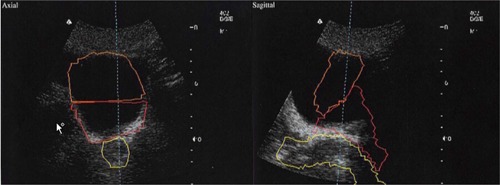
BAT template alignment. The BAT system is forced to align the cross sections of the volumes (rectum, bladder, and prostate bed) to the ultrasound image according to the CT shifts on the particular day.

The first three patients underwent treatment with BAT ultrasound localization alone. The CT shifts were calculated without any change in the ultrasound alignment. The variability appeared higher than expected (higher than 10 mm on some treatment days). This was thought to be related to difficulties in aligning the postoperative anatomy, which is more difficult to visualize in comparison to the definitive setting. To rectify this problem, special templates for BAT alignments were created during the first week of treatment for every patient, based on CT shifts calculated in real time (with the patient in the treatment position). The shifts from the CT were programmed into the BAT localization system with the 3D representation of a template (Fig. 2). The goal of using a template was to create a BAT alignment that corresponds to the prostate bed position the same as the position during CT simulation. Once a template was created, it was used at every subsequent BAT alignment for the given patient. The template was expected to help the therapists find the relative location of the treatment‐planning structures with respect to the patient anatomy in the ultrasound image.

All shifts were recorded as isocenter shifts (opposite to table shifts). The signs of the shifts were chosen in the following way: (1) positive shifts were in posterior, left, and inferior directions; (2) negative shifts were in the anterior, right, and superior directions.

In our statistical analysis the following parameters were used to quantify our results: (1) the absolute CT shift, (2) the difference between the BAT and the CT shift, (3) the absolute difference between the BAT and CT shift, and (4) a parameter that we defined as “improvement.” Each of these quantities was averaged over a number of observations for each patient. Each parameter was calculated separately in the AP, longitudinal, and lateral directions. The days on which the templates were created were excluded from the statistical analysis. Statistical significance was calculated using a correlated two‐tailed *t*‐test.

The “absolute CT shift” is defined as the magnitude of the actual shifts for a particular patient. The absolute CT shift is equal to the initial displacement of the target from its ideal position (i.e., interfraction target motion and setup error).

The “difference between the BAT and the CT shift” (difference=BAT−CT) represents the systematic error between the CT and the BAT shifts. The systematic errors represent tendencies in BAT alignment. This information is meaningful and is used to improve the BAT localization (i.e., residual shift after BAT alignment). The residual shift is assumed to be a normal distribution; therefore, its average value for a particular patient along with the standard deviation can be used to estimate how often the target is within a certain margin.

The “absolute difference between the CT and BAT shift” (abs. difference=|BAT−CT|) is a characterization of the discrepancy between the CT and BAT shift. Averaged over the number of observations, this quantity represents both the systematic and random errors (i.e., the absolute residual shift after BAT alignment).

“Improvement” illustrates the role of BAT localization assuming that the CT shifts are ideal and is defined by the following equation:

(1) improvement=|CT shift|−|BAT shift−CT shift|

**Figure 3:**
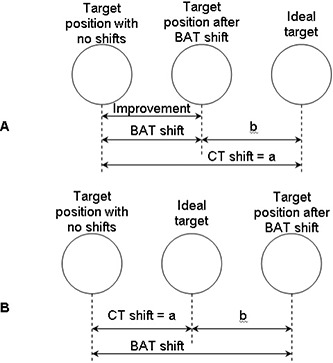
Definition of the parameter “improvement”: (A) when the BAT shift is in the correct direction but is smaller than the CT shift, the improvement is equal to the BAT shift (see |a|−|b| in A); (B) when the BAT shift is greater than the CT shift, the improvement is still defined as |a|−|b|, but it is no longer equal to the BAT shift.

The CT shifts also demonstrate setup error and target motion if no daily localization was performed (in Fig. 3, parameter *a*). The difference between the CT shift (i.e., ideal position) and the BAT shift represents the discrepancy between BAT localization and the ideal position (in Fig. 3, parameter *b*). Improvement is defined as the difference between *a* and *b* (Fig. 3), which represents the effect of BAT localization (beneficial or detrimental). When the difference between *a* and *b* is greater than zero, BAT localization improves the target positioning. A difference of less than zero demonstrates the BAT localization system is detrimental and worsens localization.

## III. RESULTS

A total of 90 image comparisons (CT versus ultrasound) were recorded for nine patients. Comparisons for each patient are presented separately in the AP, lateral, and longitudinal directions (Table 1).

**Table 1:** Comparison between CT and BAT localization

Patient#	Template (Y/N)	Number of observations	Average initial target displacement based on CT (mm) (SD^*^)			Average residual shift with sign after BAT (mm) (SD^*^) [p value]			Average absolute residual shift after BAT (mm) (SD^*^)			Average improvement (mm) (SD^*^) [p value]		
			AP	Lat	Long	AP	Lit	Long	AP	Lad	Long	AP	Lit	Long
1	N	13	4.3 (1.7)	3.7 (2.9)	5.2 (2.2)	−3.9(4.3)	3.9 (3.5)	−3.1(3.6)	4.1 (4.1)	4.8 (21)	4.1 (2.5)	0.2 (4.6)	−1.0(4.1)	1.1 (2.9)
						[0.006]	[0.002]	[0.008]				[0.859]	[0.388]	[0.208]
2	N	13	2.8 (2.2)	3 3 (1 3)	3.5 (3.3)	−3.3(6.0)	3.6 (3.7)	2.5 (4.3)	5.8 (3.5)	4.0 (3.3)	4.1 (2.7)	−3.0(2.9)	−0.6(3.7)	−0.6(3.0)
						[0.074]	[0.004]	[0.054]				[0.003]	[0.543]	[0.509]
3	N	9	2.8 (1.6)	1.8 (0.9)	2.2 (1.7)	−3.6(5.4)	1.2 (3.2)	2.7 (3.9)	5.8 (2.5)	2.8 (1.8)	3 6 (2.9)	−3.0(3.0)	−0.9(2.0)	−1.4(3.1)
						[0.080]	[0.308]	[0.070]				[0.016]	[0.194]	[0.206]
4	Y	6	8.0 (3.7)	6.0 (1.8)	4.8 (2.7)	4.3 (3.2)	−2.9(1.9)	0.3 (4 5)	4.5 (2.8)	3.2 (1.4)	3.3 (2.6)	3 5 (4 3)	2.8 (2.1)	1.5 (4.1)
						[0.022]	[0.014]	[0.876]				[0.102]	[0.022]	[0.413]
5	Y	7	7.3 (2.9)	5.3 (1.9)	2.4 (1.9)	−4.6(3.5)	−1.4(3.9)	2.6 (3.0)	5.0 (2.9)	3.3 (2.2)	3.0 (14)	2.3 (3.7)	2.1 (3.2)	−0.6(3.3)
						(0.013)	[0.373]	[0.061]				[0.146]	[0.140]	[0.656]
6	Y	10	3.7 (2.3)	2.1 (1.5)	2.3 (1.4)	−2.7(3.3)	−1.3(2.8)	0.0 (2.3)	3.4 (2.4)	2.5 (1.6)	1.8 (1.3)	0 2 (3.9)	−0.5(1.7)	0.5 (2.4)
						[0.027]	[0.163]	[0.968]				[0.855]	[0.417]	[0.545]
7	Y	10	5.2 (4.5)	2.6 (1.8)	1.9 (1.9)	0.2 (1.7)	0.7 (2.1)	0.2 (2.5)	1.3 (1.0)	l.9 (1.0)	1.8 (1.6)	3.9 (4.7)	0 8 (1.8)	0.1 (3.1)
						[0.735]	[0.348]	[0.805]				[0.029]	[0.217]	[0.904]
8	Y	11	2.1 (1.0)	2.4 (1.1)	2 9 (1.9)	0.6 (3.0)	−1.5(1.4)	1.6 (2.4)	2.5 (1.7)	1.7 (1.1)	2.0 (2.1)	−0.4(1.7)	0 6 (0.8)	0.9 (2.3)
						[0.553]	[0.006]	[0.059]				[.496]	0.031	[0.202]
9	Y	11	9.4 (2.3)	1.6 (0.7)	2.1 (1.4)	2.9 (1.7)	−1.6(2.0)	0.2 (3 3)	3.1 (1.3)	2.2 (1.2)	27 (1.6)	6 3 (2 0)	−0.5(1.2)	−0.7(2.6)
						[0.000]	[0.024]	[0.815]				[0.000]	(0.264)	[0.617]

^*^ Standard Deviation

The average absolute CT shift of the target (i.e., interfraction motion) was relatively small in the lateral and longitudinal directions. The CT shifts averaged over all patients were 3.2 mm (range of patient averages 1.6 mm to 6.0 mm) in the lateral direction and 3.0 mm (range of patient averages 1.9 mm to 5.2 mm) in the longitudinal direction. In the AP direction, the CT shifts were more substantial, with an average displacement of 5.1 mm (range of patient averages 2.1 mm to 9.4 mm). The largest single target displacement in the AP direction was 16.9 mm. A careful examination of the localization CT scans showed that the daily variation in the filling of both the bladder and the rectum was significant, resulting in shift of the prostate bed in the AP direction with minor rotation causing lateral and longitudinal shifts.

The residual shift was reduced when a template was implemented (after the first three patients). When using a template (patients 4 to 9), the residual shifts ranged from 0.2 mm to 4.6 mm (AP), 0.7 mm to 2.9 mm (lateral), and 0.2 mm to 2.6 mm (longitudinal), while for the nontemplate patients (patients 1 to 3), the residual shift was higher [(3.3 mm to 3.9 mm (AP), 1.2 mm to 3.9 mm (lateral), and 2.7 mm to 3.1 mm (longitudinal)]. The template minimized random errors in BAT alignments by reducing the standard deviations of the residual shifts over the course of treatment (especially in the AP direction: nontemplate from 4.3 mm to 6 mm; template from 1.7 mm to 3.5 mm). The template also reduced the systematic error (average residual sifts) for 4 of 6 template patients. For patients 4 and 5 the rectal filling on the day when the templates were created was different than the rectal filling during the rest of the treatment, resulting in high systematic errors in the AP direction (4.3 mm and 4.6 mm). This suggests that for some patients a second template may be needed during the course of treatment.

The template reduced the total errors (including systematic and random errors) by reducing the average absolute residual shifts (template: AP 1.3 mm to 5.0 mm, lateral 1.7 mm to 3.2 mm, and longitudinal 1.8 mm to 3.3 mm; nontemplate: AP 4.1 mm to 5.8 mm, lateral 2.8 mm to 4.8 mm, and longitudinal 3.6 mm to 4.1 mm).

**Figure 4:**
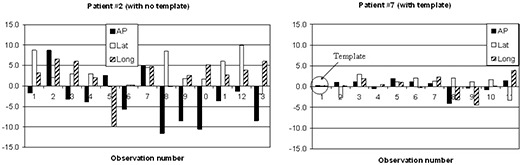
Daily residual target shift after BAT localization. One patient from each group (with and without using a template) is presented.

Figure 4 illustrates the benefit of having a BAT ultrasound template. BAT alignments for patient 7 were based on a template, while no template was used for patient 2. The residual shifts for patient 2 were much higher, exceeding 10 mm in several instances. For patient 7, all residual shifts were within 5 mm, and most of them were within 3 mm, which is similar to the uncertainty of the CT shifts. Patient 5 had significant day‐to‐day variations in rectal filling resulting not only in an anterior displacement of the prostate bed, but also in change of its shape (or rotation of the prostate bed). In such cases it is impossible to perfectly align the planning target to the daily target using linear shifts only. A good alignment in the inferior region (bladder neck) of the target will ultimately lead to misalignment in the superior region and vice versa. The alignment then becomes a matter of clinical decision made by the treating therapist on a daily basis, which is guided by the physician. This issue was the main reason for the results seen for patient 5 compared to the other template patients.

Target motion in the AP direction is a serious obstacle when treating post‐prostatectomy patients. A margin of 8 mm in the AP direction is used at our institution. Based on the residual shifts for nontemplate patients, the target was within the margin in less than 83% of the cases (patient 1: 82.7%; patient 2: 75.3%; patient 3: 77.6%), while other template patients were all covered in greater than 83% (range 83.4% to 99%). The low percentage values for patients 4 (87.6%) and 5 (83.4%) were mostly due to the high systematic component of their residual shifts. However, the standard deviations (random component) of the residual shifts for these patients were considerably lower than the standard deviations for the nontemplate patients, meaning that further improvement was possible, as mentioned above.

**Figure 5:**
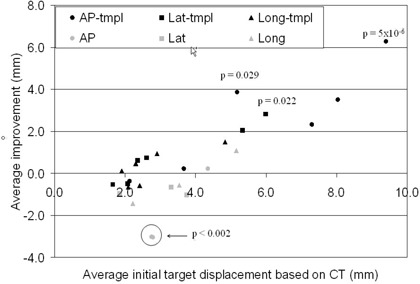
Average improvements for all nine patients. Some *p* values are also presented.

Improvement is limited by the initial displacement of the target. For example, if the target happens to be in a perfect position before the daily BAT alignment, further improvement is impossible. The average improvements in each direction for all nine patients are presented as a function of the initial target displacement in Fig. 5. The figure illustrates that in both the longitudinal and in the lateral directions, the improvements are small when the initial target displacements are small (less than 4 mm). In these directions, improvements are either positive or negative for the template patients, and mostly negative (meaning deterioration of the positioning) for the nontemplate patients. These values are insignificant when the initial displacement is small. However, in the AP direction a statistically significant deterioration in positioning was demonstrated for two of the three patients treated without the aid of a template even at small initial displacements of the target (average improvement of −3.0mm with p<0.002).

In all cases where the initial target displacement was greater than 5 mm, the improvements were positive for the template patients. Statistically significant improvements increase as the target displacement increases. The CT‐based template is most crucial for the BAT alignment in the AP directions. The maximum average improvement of a template patient is also in the AP direction and has a value of 6.3 mm $\left(p=5\times 10^{-6}\right)$.

## IV. DISCUSSION

Radiation therapy with or without androgen deprivation following a radical prostatectomy is considered for patients with pathologic T3 disease, positive surgical margins, or a slowly rising PSA profile. Contrary to the treatment of prostate cancer in the definitive setting, the target volume in the postoperative setting is not well‐defined. The most common sites of clinical failure are the peri‐anastomotic site, bladder neck, retrovesical space, and, rarely, the regional lymph nodes.^(21)^ Similar to patients treated in the definitive setting, precision radiotherapy requires reducing daily uncertainties in order to ensure target coverage while minimizing radiation exposure to critical surrounding structures.

In this study, the interfraction motion was measured using the average CT shifts over the course of treatment for each patient (called initial target displacement). As mentioned above, the uncertainty of the CT shifts was 2.5 mm. The average interfraction motions in the lateral, longitudinal, and AP directions were 3.0 mm, 3.2 mm, and 5.1 mm, respectively; these results are similar to the values in the treatment of definitive patients.^(^ ^5^ ^,^ ^22^ ^,^ ^23^ ^)^ The CT scans were then used to evaluate the accuracy of BAT ultrasound localization in the postoperative setting. With the lack of a clearly defined target, such as the prostate, systematic errors in BAT ultrasound alignment were found. ImplnXentation of a template BAT ultrasound based on CT significantly reduced systematic errors and led to improved target localization, especially in the AP direction.

Specifically looking at the AP direction, an 8‐mm margin was added to the clinical target volume to account for daily uncertainties. The target of the nontemplate patients was fully within this margin for between 75.3% and 82.7% of the scans, while the aid of the template improved the tumor coverage to 83.4% to 99%. Actually, all but two of the patients treated with the aid of the template exhibited greater than 94.5% coverage. The fact that the target was fully covered in 87.6% of the treatments for patient 4, and 83.4% of the treatments for patient 5, suggests that probably a planning tumor volume (PTV) margin of greater than 8 mm is needed. However, positioning errors for these patients were mostly due to their high average residual shifts, whereas the standard deviations of the residual shifts were considerably lower than the standard deviations for the nontemplate patients. The implication is that the discrepancies between the BAT and CT shifts are primarily systematic for the template patients, and therefore these discrepancies may be reduced by creating a second template during the course of treatment. If the template technique fails to reduce the systematic errors, then an increased patient specific margin should be considered

The reduction of both systematic and random discrepancies for the template patients resulted in lower absolute residual shifts after BAT alignment (Table 1. This applies to all template patients except patients 4 and 5, which, for example, had average absolute residual shifts in the AP direction of, respectively, 4.5 mm and 5.0 mm. However, these residual shifts were primarily due to systematic differences between the BAT and CT shifts, and the systematic differences could be decreased as previously discussed. In the future, when more data on template assisted alignments is collected, further analysis could be performed, in order to correlate the magnitude of the residual shifts with patient specific parameters, such as weight, for example. Such a study would help develop guidelines on choosing a patient specific PTV margin.

Chinnaiyan et al.^(24)^ recently reported a similar series using an optically guided 3D ultrasound guided localization. They reported average absolute ultrasound shifts of 5±4mm,3±3mm,3±4mm, over the entire course of treatment for 6 patients treated in the postoperative setting in the AP, lateral, and CC directions, respectively. These values are similar to 10 patients treated in the definitive setting. In their study, the bladder neck was used as the primary reference to minimize systematic errors. In addition, a rectal balloon was used on a daily basis for internal immobilization. In the same study the ultrasound shifts were compared with shifts based on portal films and digitally reconstructed radiographs from the time of simulation. However, the purpose of this comparison was not to evaluate the accuracy of the ultrasound shifts but only to differentiate the setup errors from the actual internal target motion. Although steps were taken to limit these uncertainties with the use of the rectal balloon, better daily imaging (CT) could be used to evaluate the ultrasound shifts. The rectal balloon has also been shown to reduce the volume of rectum receiving a significant radiation dose,^(25)^ but this is counterbalanced by the discomfort of the patient, the daily reproducibility of the balloon, and the potential hypoxia caused by the pressure of the balloon, which could alter the effectiveness of the radiation.^(26)^

This study proposes a localization technique based on CT and ultrasound image guidance that reduces uncertainties of organ motion and setup error in the postoperative setting. However, clearly there are serious concerns when considering reduction of treatment margins in cases where the target volumes are not well‐defined. In addition, there are also concerns whether the target volumes move in relationship to each other and whether uniform margins are appropriate. In the definitive setting, similar issues exist but are typically less significant. The distal seminal vesicles relative to the prostate move more than the proximal seminal vesicles. Furthermore, the movement of the lymph nodes relative to the prostate and seminal vesicles is unknown. In the postoperative setting, the bladder neck and retrovesical space move relative to the filling of the bladder and rectum, although the peri‐anastomotic site does not. In addition, there is the issue of the movement of the pelvic lymph nodes. Currently, we are using serial CT scans in the definitive and postoperative setting to further evaluate these issues. Daily imaging to reduce daily uncertainties should be beneficial, but further studies are necessary to ensure adequate margins to account for target motion and whether immobilization of the tumor bed is necessary and/or useful.

## V. CONCLUSIONS

The presented results suggest that when the initial displacement of the prostate bed is greater than 4 mm, daily ultrasound localization can be improved with the aid of a CT‐based template. When the initial target displacements are smaller than 4 mm, the presented technique is neither beneficial nor detrimental. Patient positioning performed using ultrasound alignments without the aid of a CT‐based template may potentially worsen the localization of the prostate bed.
